# Challenges and opportunities of bioprocessing 5-aminolevulinic acid using genetic and metabolic engineering: a critical review

**DOI:** 10.1186/s40643-021-00455-6

**Published:** 2021-10-13

**Authors:** Ying-Chen Yi, I-Tai Shih, Tzu-Hsuan Yu, Yen-Ju Lee, I-Son Ng

**Affiliations:** Department of Chemical Engineering, National Cheng Kung University, Tainan, 70101 Taiwan

**Keywords:** 5-aminolevulinic acid, Heme, Bioprocessing, metabolic engineering, C4 and C5 pathway, Photodynamic therapy

## Abstract

5-Aminolevulinic acid (5-ALA), a non-proteinogenic five-carbon amino acid, has received intensive attentions in medicine due to its approval by the US Food and Drug Administration (FDA) for cancer diagnosis and treatment as photodynamic therapy. As chemical synthesis of 5-ALA performed low yield, complicated processes, and high cost, biosynthesis of 5-ALA via C4 (also called Shemin pathway) and C5 pathway related to heme biosynthesis in microorganism equipped more advantages. In C4 pathway, 5-ALA is derived from condensation of succinyl-CoA and glycine by 5-aminolevulic acid synthase (ALAS) with pyridoxal phosphate (PLP) as co-factor in one-step biotransformation. The C5 pathway involves three enzymes comprising glutamyl-tRNA synthetase (GltX), glutamyl-tRNA reductase (HemA), and glutamate-1-semialdehyde aminotransferase (HemL) from α-ketoglutarate in TCA cycle to 5-ALA and heme. In this review, we describe the recent results of 5-ALA production from different genes and microorganisms via genetic and metabolic engineering approaches. The regulation of different chassis is fine-tuned by applying synthetic biology and boosts 5-ALA production eventually. The purification process, challenges, and opportunities of 5-ALA for industrial applications are also summarized.

## Introduction and development of 5-ALA

5-aminolevulinic acid (5-ALA), an endogenous non-proteinogenic five-carbon amino acid, is the indispensable intermediate which involves in the tetrapyrrole biosynthesis and is the first compound from the porphyrin synthesis to heme. 5-ALA as the precursor of heme, chlorophylls, and vitamin B_12_ significantly affects the cell growth and metabolic flux. 5-ALA has been tested and applied as a prodrug for leukemia cells treatment (Malik and Lugaci 1987). In this therapy, 5-ALA was observed in the porphyrin metabolism, which 5-ALA converted to photosensitizer protoporphyrin IX (PPIX) and showed fluorescence signal when 5-ALA was accumulated inside the cell. PPIX would produce reactive oxygen species (ROS) under appropriate wavelength and causes cell damage by generating singlet oxygen. With PPIX accumulation, the tumor cell is visualized with emission of fluorescence. 5-ALA-induced photodynamic therapy and diagnosis had been reported since 1990 (Kennedy et al. 1990) and was approved by FDA in 2017.

On the other hand, therapy with 5-ALA not only possesses potential for topical, rapid and effective application but is also the non-toxic to human body (Wild et al. 2005). Therefore, 5-ALA has more advantages than using chemotherapy and surgeries (Peng et al. 1997a). When the reduced ferrochelatase activity in the tumor cells, the excess PPIX leads to formation of reactive oxygen species and thereby cell apoptosis when exposed to suitable wavelength (Peng et al. 1997b). Besides, 5-ALA can be used to monitor the difference between tumor and normal tissue by time (Krieg et al. 2002; Yang et al. 2015).

In addition to the application in medicine, 5-ALA has also been employed in the agriculture as it is harmless and belongs to biodegradable herbicide and insecticide, growth-promoting factor, thus enhancing the productivity of crops (Hotta et al. 1997). When plants are treated with high concentration of 5-ALA and exposed to light, the ROS produced by excess PPIX will oxidize unsaturated fatty acids, thereby damaging plants. The same mechanism was also observed when proper 5-ALA concentration was used for biodegradable insecticide (Sasikala et al. 1994; Sasaki et al. 2002). Although the ROS was generated at a low level under the normal condition, the balance was disturbed by environmental stress, such as salt, temperature, and drought, which would rapidly increase ROS thus to cause oxidative damage to lipids, proteins, and nucleic acids in the cell. As a key precursor in the biosynthesis of porphyrins, 5-ALA has been considered to increasing the yield of foliage and root. Previous studies showed that the yield and quality of plants and crops could be improved by 5-ALA (Hotta et al. 1997; Dolmans et al. 2003; Liu et al. 2013). As a conclusion, 5-ALA is able to promote seedling growth of plants by raising chlorophyll content and net photosynthetic rate to prevent stress damage (Naeem et al. 2012; Liu et al. 2014).

Till now, six methods of chemical synthesis for 5-ALA have been mentioned. 5-ALA can be synthesized from levulinic acid, 2-hydroxypyridine, furfural, furfurylamine, tetrahydrofurfurylamine, and succinic acid (Sasaki et al. 2002). Above all, the photo-oxidation of furfurylamine is regarded as the most practical method. However, with the awareness of environmental protection and sustainability, strategies related to biosynthesis of 5-ALA have attracted more attentions. Two main metabolic pathways, C4 and C5 pathway, for the biosynthesis of 5-ALA from the precursors on tricarboxylic acid (TCA) cycle in organisms have been reported (Fu et al. 2010; Kang et al. 2004, 2017). Interestingly, the C4 pathway is present in birds, mammals, yeast, and purple non-sulfur-photosynthetic bacteria, while the C5 pathway exists in algae, higher plants, and many bacteria, including *Escherichia coli* and archaea (Yang et al. 2016). In the C4 pathway, 5-ALA is derived from the condensation of succinyl-coenzyme A (succinyl-CoA) and glycine by the 5-ALA synthase (ALAS, encoded by the *hem*A gene) (Sasaki et al. 2002). On the other hand, 5-ALA formation involves three sequential enzymatic reaction catalyzed by glutamyl-tRNA synthetase, glutamyl-tRNA reductase, and glutamate-1-semialdehyde aminotransferase (encoded by *glt*X (Huang and Wang 1986; Schneegurt and Beale 1988), *hem*A (Li et al. 1989; Schauer et al. 2002), and *hem*L (Jahn et al. 1992; Wang et al. 1984) gene, respectively) from TCA cycle intermediate, glutamate in C5 route. The C4 pathway uses a unique 5-ALA synthase (ALAS) as single-step reaction is easier for regulation than that applies the C5 pathway, even most prokaryotes originally obtain this pathway. But C5 pathway is not limited or restricted by using glycine as the precursor. In the view of green, sustainable, and highly efficient production of chemicals with appropriate organisms, for example, *Escherichia coli*, is more favorable.

Sasaki et al. has reviewed the biosynthesis and application of 5-ALA in 2002 (Sasaki et al. 2002). They have pointed out the strategies and affecting factors for 5-ALA production in microorganisms. The recent advances in synthetic biology have been explored for 5-ALA biosynthesis. The microbial production of 5-ALA through biological and genetic approaches and challenges were also discussed recently (Kang et al. 2017). Herein, we will introduce the determinant factors and novel strategies for 5-ALA bioproduction via C4 and C5 pathway by genetic, synthetic, and metabolic approaches in the last decade. Moreover, the regulation between 5-ALA accumulation and heme, purification, challenges, and prospective of 5-ALA for industry are also discussed.

## Production of 5-ALA via C4 pathway in *Escherichia coli*

In previous study, *hemA*^C4^ gene fragment was mostly amplified from purple non-sulfur bacteria, such as *Rhodobacter sphaeroides* and *Rhodobacter capsulatus*. ALAS is a PLP-dependent homodimeric enzyme which could catalyze the decarboxylation as well as the deprotonation (Liu et al. 2014). Co-factor PLP firstly binds to an active site lysine and therefore changes conformation endowing ALAS with ability to catalyze the condensation reaction. Next, glycine binds to ALAS-PLP complex accompanied with deprotonation and forms a quinonoid intermediate (Stojanovski et al. 2014). Subsequently, succinyl-CoA reacted with the quinonoid intermediate to generate an unstable compound. Active site histidine would catalyze the decarboxylation after release of CoA. Structure simulation and analysis have unveiled that the lysine and histidine residue at K248 and H142 of RcALAS are responsible for the affinity to co-factor and substrates. Stojanovski et al. have taken advantage of simulation to illustrate that the substrate specificity of ALAS was attributed to the conformational hindrance if ALAS-PLP complex bounded to amino acid except for glycine (Stojanovski et al. 2014).

As an enzyme belongs to α-oxoamine synthase (AOS) family, ALAS adapts asparagine instead of histidine and phenylalanine which are widely observed at analogous site in the structure. The unusual amino acid usage is assumed that histidine and phenylalanine may impede the 5-ALA release and even result in balance shift to reverse reaction (Stojanovski and Ferreira 2015).

### Gene selection and enzyme activity

Choosing proper proteins to redirect the carbon flux to the target chemicals is highly significant because the enzyme from different organisms also diverged considerably in its specific activity, kinetic constants, and optimal conditions. An extremely active enzyme can catalyze the reaction more rapidly as well as transform the substrates to products more efficiently. 5-Aminolevulinic acid synthase (ALAS), a key enzyme of the C4 pathway, is encoded by *hem*A gene from different organisms, such as *Agrobacterium radiobacter* (Fu et al. 2010), *Bradyrhizobium japonicum* (Choi et al. 1999; Jung et al. 2004), *Rhodobacter capsulatus* (Kang et al. 2004; Lou et al. 2014; Yu et al. 2020)*, Paracoccus* (Sato et al. 1985), *Rattus norvegicus* (Nakakuki et al. 1980), *Rhodobacter sphaeroides* (Kang et al. 2011a, b; Shih et al. 2021; Tan et al. 2020a; Tran et al. 2019), *Rhodopseudomonas palustris* (Ong et al. 2013; Zhang et al. 2013; Liu et al. 2016), and *Saccharomyces cerevisiae* (Volland and Felix 1984). The ALAS from different organisms used for 5-ALA production via C4 pathway in the genetic *E. coli* strains are summarized in Table 1. Among all, the specific activities of ALAS from *R. sphaeroides*, *A. radiobacter*, *R. capsulatus,* and *R. palustris* were 0.117, 0.151, 0.198, and 0.924 µmol/min/mg, respectively (Lou et al. 2014), while Meng et al. reported that the ALAS from *Laceyella sacchari* possessed the highest activity of 7.8 µmol/min/mg (Meng et al. 2015). Although the reaction system and approach from separate group would lead to different calculation, the comparison of ALAS activity from diverse strains is still useful. The activity and characteristic of enzymes have also been organized in BRENDA database (www.brenda-enzymes.org). From the database, the ALAS from *R. palustris* possesses higher activities than other organisms, which made it a promising candidate for high-efficient 5-ALA yield. Based on this concept, Zhu et al. have co-expressed *R. palustris* ALAS, catalase, and superoxide dismutase in *E. coli*, and finally achieved the highest 5-ALA production in record as 11.5 g/L in a 5-L bioreactor using fermentation (Zhu et al. 2019).

**Table 1:** Enzymatic activity of ALAS from different species

Species	P/C^a^	Specific activity (µmol/min/mg)	Ref
*Rhodobacter capsulatus*	P	0.19	Lou et al. (2014)
	C	0.05	
*Rhodobacter sphaeroides*	P	0.11	
	C	0.03	
*Agrobacterium radiobacter*	P	0.15	
	C	0.02	
*Rhodobacter sphaeroides*	P	3.3	Meng et al. (2015)
*Rhodopseudomonas palustris*	P	4.4	
*Geobacillus thermoglucosidasius*	P	1.4	
*Laceyella sacchari*	P	7.8	
*Pseudomonas alcaliphila*	P	0.58	

^a^ Indicates the enzyme condition at 37 °C and pH 7.5 for characterization. P means the ALAS was purified and C means that crude enzyme was used

### Codon optimization of ALAS

Codon optimization of ALAS is favorable to reach higher protein expression level, protein structures, and specific activity to further obtain higher 5-ALA production. In the central dogma, protein expression is the final step of several complicated processes, including regulation at the level of transcription, mRNA turnover, protein translation, and post-translational modifications leading to the formation of the stable and functional protein. Although there are only twenty amino acids in protein sequences, the codons specified for all the amino acids are sixty one, i.e., codon usage bias. Thus, the regulation at the level of transcription is highly relative to the codon usage bias (Kane 1995). For example, the heterologous protein expression showed the lower protein yield due to the rare codon usage bias or the insufficient of transfer RNA (tRNA) as the native codon is used. Consequently, for most sequenced genomes, synonymous codons are not equal-frequently used, as called “non-optimal codons,” leading to the low protein level due to the limiting available cognate tRNAs in the expression host. Kane also discussed the effect of rare codons for heterologous protein expression in *E. coli*, reporting that some codons would cause translational errors (Kane 1995). Angov reviewed the effect of codon bias on mRNA structure and gene translation, which further regulated the protein expression levels (Angov 2011). The approaches for codon usage analysis and optimization tools were also provided by using a software for calculation of codon adaptation index (CAI), which was an empirical measure of protein expressivity (Fuglsang 2003). Menzella evaluated “one amino acid one codon” and “codon randomization” for codon optimization, suggesting that more protein expression was observed from the sequence with codon randomization (Menzella 2011). Accordingly, the *hem*A gene from *S. arizona* was expressed after codon optimization and enhance 5-ALA accumulation in *E. coli* via C5 pathway (Zhang et al. 2015, 2018a). Li et al. also expressed codon-optimized ALAS from *S. cerevisiae* in *E. coli* and improved 5-ALA titer via C4 pathway (Li et al. 2016). Furthermore, Yu et al. showed the expression of codon-optimized *R. capsulatus* ALAS with chaperone would obtain more soluble ALAS and further produce high level of 5-ALA (Yu et al. 2020). However, the overly excessive protein expression level leads to the formation of insoluble products sequestered in inclusion bodies which hinder the function of proteins (Sørensen and Mortensen 2005). As the result, the modification of codon for regulation of optimal expression level should be the priority instead of reaching the highest protein production. Besides, the pRARE plasmid carrying tRNAs recognizing rare codons was also reported to be effective to improve heterologous protein expression (Burgess-Brown et al. 2008; Effendi et al. 2020). Moreover, the Rosetta(DE3) strain was acquired by introducing pRARE into *E. coli* BL21(DE3) for assisting the transcription. The ALAS from *R. sphaeroides* and *A. radiobacter* was expressed under T7 promoter in Rosetta(DE3) and more 5-ALA was reached with higher enzyme activity than that in BL21(DE3) (Fu et al. 2007, 2010; Yang et al. 2013). The activities of ALAS from *A. radiobacter*, *R. sphaeroides* and *R. capsulatus* were compared in Rosetta(DE3). Among the enzymes, ALAS from *R. capsulatus* showed the highest activity and 8.8 g/L of 5-ALA titer was achieved (Lou et al. 2014). Besides, GenScript company (https://www.genscript.com/tools/rare-codon-analysis) provides the online software, while Integrated DNA Technologies® supports codon optimization tool for DNA synthesis which meets the goal to obtain higher expression level.

### Transporter and chaperone

As high concentration of 5-ALA is toxic to *E. coli*, it is necessary to avoid the 5-ALA accumulation in the cell. Even though cell can drain the metabolism out of the cell spontaneously, the cell still requires transporter to accelerate the export of 5-ALA. Several amino acid transport systems are identified in *E. coli,* while the efflux mechanism of 5-ALA is still an enigma (Zhu et al. 2020). Auspiciously, the structure of 5-ALA is similar to glycine; thus, an amino acid exporter would solve the problem. RhtA is capable of translocating a variety of amino acids and related compounds, such as dipeptide and amino acid analogs, and has been reported extremely powerful for different amino acid excretion (Diesveld et al. 2009). Yang et al. accomplished 5-ALA up to 14.7 g/L via application of RhtA in *C. glutamicum* (Yang et al. 2016). The YeaS is a leucine exporter, which also encodes to be RhtB transporter family. The over-expression of *yeaS* results in resistance of cells to leucine analogs, glycyl-L-leucine dipeptide, and other amino acids. However, the efficiency of YeaS is much less than RhtA (Kang et al. 2011a, b). Therefore, RhtA is an efficient transporter for 5-ALA till now.

Chaperone which assists the folding of protein is a critical part to be deliberated for the insoluble aggregates of the foreign protein expressed in *E. coli*. Recent report has demonstrated the optimal chaperone is powerful to improve the functional expression of recombinant horseradish peroxidase (Yang et al. 2019a, b). Considering the translational effect of ALAS and preventing the inclusion bodies forming is crucial for ALA production. The GroELS system includes GroEL and GroES, while DnaK system consists of DnaK, DnaJ, and GrpE are the most eminent chaperone systems (de Marco et al. 2007). GroEL is a cylindrical complex of double ring of subunits that composes of an ATP-binding domain, an intermediate hinge domain, and an apical domain in the three-dimensional structure as a protein-folding machinery in *E. coli* (Hayer-Hartl et al. 2016). The previous research has applied GroEL chaperone to enhance 5-ALA production with RcALAS from 1.2 g/L to 3.6 g/L due to the improvement of the soluble protein (Yu et al. 2020). DnaK, DnaJ, and GrpE are the members of Hsp70 chaperone family. At first, DnaK prevents and repairs the thermally induced protein damage in *E. coli*. Moreover, DnaK also binds with various abnormal proteins and protects RNA polymerase from thermal inactivation. On the other hand, DnaJ overturns the aggregation of inactive protein and target DnaK to the substrate. Shih et al. compared the 5-ALA production with the assistance of GroELS and DnaKJ, respectively (Shih et al. 2021). Although DnaK has been reported to refold other inactive proteins, the efficiency is not observable in 5-ALA production (Xie et al. 2003; Yang et al. 2019a, b; Yu et al. 2020). Till now, GroE system is more beneficial to ALAS folding and leading higher 5-ALA production. Meanwhile, it is worth mentioning that high 5-ALA concentration in medium may harm cell via generating ROS. Therefore, expressing catalase and superoxide dismutase is a promising strategy to enhance the tolerance of *E. coli* toward the oxidative stress caused by ROS from 5-ALA (Zhu et al. 2019). The effects of 5-ALA on cell integrity and morphology were demonstrated to deal with oxidative stress and enhance 5-ALA accumulation effectively in *E. coli*.

### Medium and trace element

Thanks to the development of genetic engineering, biosynthesis is a more feasible and efficient strategy for 5-ALA production of chemicals in recent decades. In 1996, Van der Werf and Zeikus investigated the effect of different carbon sources and amino acids on 5-ALA production (Van der Werf and Zeikus 1996). Luria–Bertani broth (LB) and Terrific Broth (TB) composed of tryptone and yeast extract have been widely used as nutrient-rich media and have been applied for 5-ALA production (Choi et al. 2008; Lou et al. 2014; Van der Werf and Zeikus 1996). However, minimum medium that includes only glucose, inorganic nitrogen source, and mineral salts as a cost-effective medium has been more favorable for 5-ALA production in recent years (Choi et al. 2008; Kang et al. 2011a, b; Zhang et al. 2015). In the early study of medium effect, concentrations of precursors (i.e., glycine and succinic acid) have the great influences on 5-ALA production in recombinant *E. coli* BL21(DE3)-pLysS and achieved 1.3 g/L (Chung et al. 2005). Lin et al. have explored that not only glucose but also xylose could serve as inhibitor of HemB. With supply of glucose and xylose, the 5-ALA production yield reached to 7.3 g/L (Lin et al. 2009). Cui et al. demonstrated that yeast extract played an important role in 5-ALA production (Cui et al. 2019). Although 12 g/L yeast extract dramatically enhanced both cell growth and 5-ALA accumulation, lower concentration of yeast extract was more feasible for industrial bioproduction.

For ion effect, zinc, copper, and cobalt ion are definitely affecting the ALAS activity (Liu et al. 2016; Lou et al. 2014). HemB would be only activated by a quite low concentration of Mn^2+^ ion. Moreover, Mg^2+^ ion even activated HemB activity (Erskine et al. 1999; Nandi et al. 1968). Thus, concentrations of Mn^2+^ and Mg^2+^ ion are the critical factors for 5-ALA production. Cui also showed that 1 g/L MgSO_4_·7H_2_O and 0.01 g/L MnSO_4_·7H_2_O were moderate for 5-ALA production in *E. coli* (Cui et al. 2019). Reducing magnesium salts and adding calcium salts could be a feasible formula for enhancing 5-ALA production, since Ca^2+^ ion showed less influence on ALAS activity. Most recently, Fe^2+^ ion could be a tricky factor for increasing 5-ALA accumulation. With 30 µM Fe^2+^ supplement, the 5-ALA titer has significantly increased in *R. sphaeroides* (Tangprasittipap et al. 2007), while addition of 1 mM Fe^2+^ showed a dramatical decline in ALAS activity (Nakakuki et al. 1980). Therefore, defining the moderate Fe^2+^ ion concentration is a promising strategy to further enhance 5-ALA biosynthesis as well.

### Gene deletion in the chromosome

As the genetic engineering tools are well developed, producing strains are no longer limited to use well-known hosts, for example, original *E. coli* BL21(DE3) or MG1655. Manipulation of several genes could be conducted to refine a strain into unique host which pushes and pulls the metabolic flux into product-oriented pathway.

**Fig. 1:**
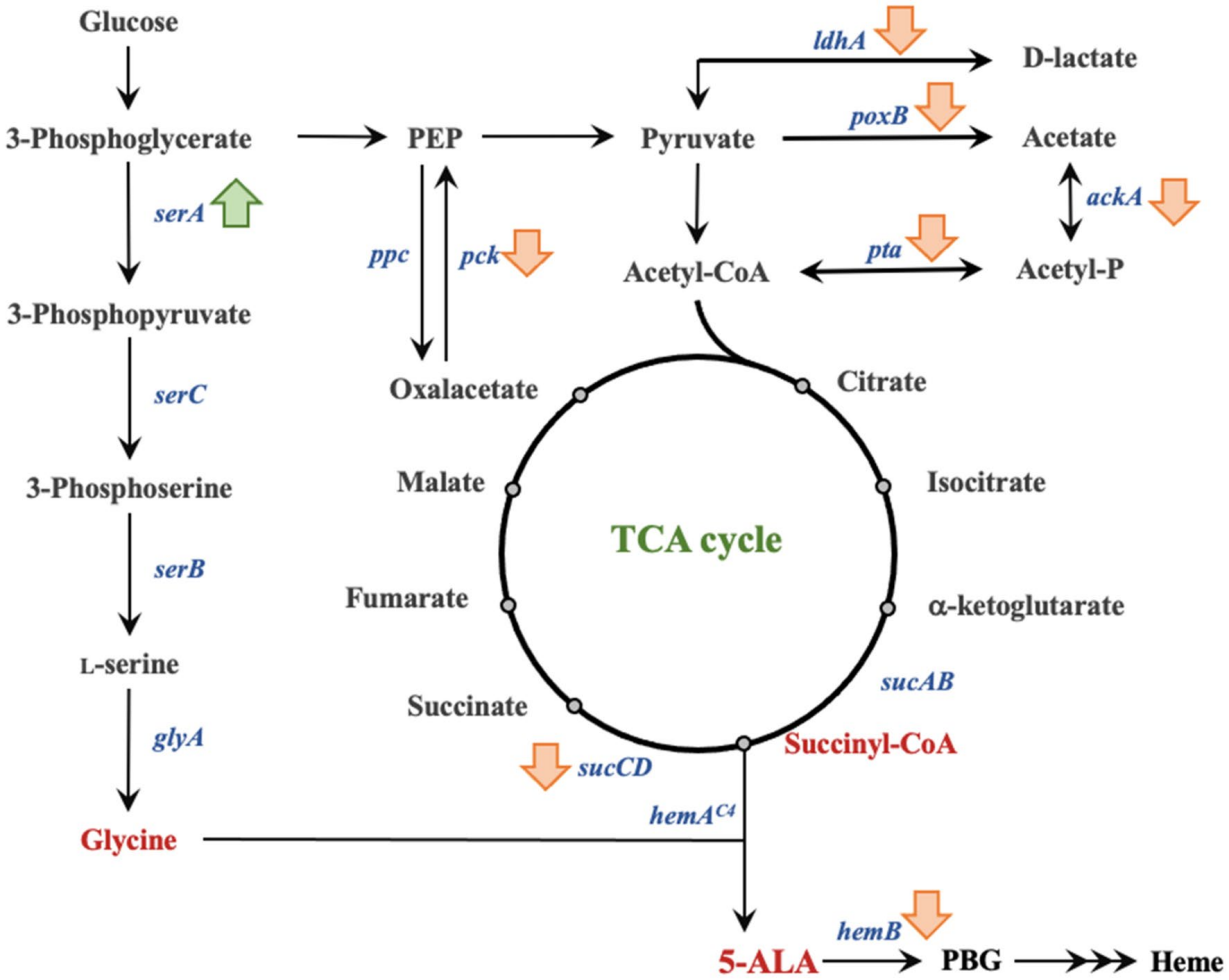
The C4 pathway for 5-ALA and heme synthesis. The up and down arrows beside the gene indicate the up- or down-regulation of the gene which can increase 5-ALA accumulation

Recently, genes deletion has become a promising strategy for further promotion of 5-ALA. Noh et al. have revealed that deletion of *sucA* leaded to lower biomass but higher 5-ALA production in *E. coli* (Noh et al. 2017). However, deleting *iclR*, a transcriptional regulator of glyoxylate-related genes (*aceBAK*), would redistribute the metabolic flux from TCA cycle to glyoxylate route but reduce 5-ALA production due to down-regulating the isocitrate dehydrogenase activity. Afterward, Ding et al. knocked out *sdhAB* and *sucCD* to block conversion of succinyl-CoA into other down-stream metabolites (Ding et al. 2017). According to the results, only deletion of *sucCD* showed a significant increase in 5-ALA titer. On the other hand, deletion of *recA* (Cui et al. 2019) and *endA* (Zhang et al. 2019) which prevented homologous recombination by the hosts thus enhanced the genetic stability. Recently, Miscevic et al. also represented 5-ALA production from glycerol in *E. coli* through metabolic engineering (Miscevic et al. 2020). The *hem*B was depressed and the *ldh*A was deleted to dissimilate carbon flux toward the TCA cycle. Afterward, the genes *sdh*A and *icl*R encoding succinate dehydrogenase complex flavoprotein subunit A and transcriptional AceBAK operon repressor were knocked out to further enhance succinyl-CoA formation in the cell. With the double mutant of *icl*R and *sdh*A, higher 5-ALA production was achieved since the succinyl-CoA could be increase by the carbon flux at the succinate node via the glyoxylate shunt (Fig. 1).

To sum up, the 5-ALA productions via C4 pathway in *E. coli* are listed in Table 2 and gene knock out is a double-edged concept. Each metabolite plays a role in the whole metabolism network. Thus, gene deletion will easily trigger the imbalance due to the metabolic flux changes and causes either poor cell growth or low productivity. The trade-off between cell growth and product titer must be considered. Also, replenishment or regeneration of crucial compounds should be a possible strategy to compensate the gene deficiency.

**Table 2:** Production of 5-ALA via C4 pathway in *Escherichia coli*

Strains	Genes for Over-expression	Strategy	Titer (g/L)	Productivity (g/L h)	Ref
BL21	*hemA* (*R. palustris*)	Addition of 45 mM glucose as an ALAD inhibitor in the late-log culture phase	5.15	0.32	Chung et al. (2005)
BL21 (DE3)	*hemA* (*R. capsulatus*)	Integrating pdxY for PLP regeneration and co-expressing ALAS with GroELS	8.21	0.23	Xue et al. (2021)
	*hemA* (*R. capsulatus*) EcGroELS	Co-expression and delay substrate addition	5.4	0.225	Yu et al. (2020)
	*hemA* (*R. capsulatus*) *coaA*^M^ (R106A), *dfp*, *coaD* (*E. coli*)	Δ*serA*^M^p::J23119, Δ*sucCD*, Δ*hemB*(ATG)::GTG	2.8	0.09	Ding et al. (2017)
	*hemA* (*B. japonicum*)	Fermenter with an addition of 30 mM glycine, 90 mM succinate and 30 mM levulinic acid	2.62	0.187	Choi et al. )^1999^)
	*hemA* (*R. sphaeroides*), rhtA	Quantification of 5-ALA simultaneously by green fluorescence protein	2.46	0.103	Tan et al. (2020a, b)
	*hemA* (*R. sphaeroides*), *aceA* (*E. coli*), *agxt* (*Homo sapiens*)	Glycine-OFF riboswitch for repression of *hemB*	0.24	0.008	Zhou et al. (2019)
BL21*(DE3) pLysS	*hemA* (*B. japonicum*)	Optimal glycine and succinic acid as 15 and 30 mM	1.30	0.05	Jung et al. (2004)
BW25113	*hemA* (*R. palustris*), *katGE*, *sodABC* (*E. coli*)	Up-regulate catalase and superoxide dismutase to combat ROS	11.5	0.52	Zhu et al. (2019)
	*hem*A (*R. sphaeroides*)	Repress *hem*B with CRISPRi and increase Suc-CoA by deleting *ldh*A, *sdh*A and *icl*R	6.93	0.32	Miscevic et al. (2020)
Rosetta(DE3)	*hemA* (*A. radiobacter*)	Short-term dissolved oxygen shock during aerobic fermentation	9.4	0.43	Yang et al. (2013)
	*hemA* (*R. capsulatus*)	Optimum pH* and temperatures of enzyme at pH 7.5 and 37 °C	8.8	0.24	Lou et al. (2014)
	*hemA* (*A. radiobacter*)	Fed-batch culture in 15-L	6.5	0.24	Fu et al. (2010)
	*hemA* (*R. sphaeroides*)	Compare the activity of ALAS in Rosetta(DE3) and BL21(DE3)	3.8	0.21	Fu et al. (2007)
MG1655	*hemA* (*R. palustris*) *hemO* (*R. palustris*)	Co-expression of ALAS isozyme HemO in 5-L bioreactor	6.3	0.35	Zhang et al. (2013)
	*hemA* (*R. sphaeroides*)	6 g/L succinate, and 0.1 mM IPTG	5.2	0.43	Van der Werf et al. (1996)

## Production of 5-ALA by regulation of C5 pathway in *E. coli*

The rate-limiting enzyme in C5 pathway is HemA catalytic reaction. HemA catalyzes the reaction that convert the glutamyl-tRNA glutamate-1-semialdehyde (GSA). Interestingly, HemA and HemL would assemble a synergistic complex to protect highly reactive compound GSA (Lüer et al. 2005). After GSA formation, HemL, a PLP-dependent enzyme (Zhang et al. 2019), could catalyze the quick reaction to convert glutamate-1-semialdehyde to 5-ALA. In recent publications, *hem*A was mostly derived from *Salmonella sp.*, while *hem*L was derived from *E. coli* for enhancing 5-ALA biosynthesis. To over-express *glt*X gene playing an intuitive way to enhance 5-ALA production, however, it may not work as expected. According to Su et al.s’ study, over-expression of *glt*X may even up-regulate *hem*B transcription and result in lower 5-ALA accumulation (Su et al. 2019). This phenomenon indicates strong feedback inhibition of *glt*X which is also controlled by the final product, heme.

### Fine-tuning heme and regulation in the pathway

In 1970, the first research of 5-ALA production through C5 pathway by *Chlorella* was published in which the carbon dioxide was chosen as the main substrate (Beale 1970). Afterward, the biosynthesis of 5-ALA has been considered as the rate-limiting step for heme biosynthesis and is regulated in organisms (Verderber et al. 1997). Heme is a porphyrin derivative and essential hemoproteins which are important for oxygen transport, eliminating ROS and transferring electrons for energy generation (Mayfield et al. 2011). The transcriptional regulation of *hem*C, *hem*D, *hem*H, *hem*A, and *hem*M genes on heme synthesis pathway was firstly examined by McNicolas et al., revealing the regulation between genes and heme availability in *E. coli* (McNicolas et al. 1997). Kwon et al. over-expressed *hem*A from *R. capsulatus* to increase 5-ALA and down-stream genes, including *hem*B, *hem*C, *hem*D, and *hem*F genes from *E. coli*, *hem*E gene from *Synechocystis* sp., and *hem*H gene from *Bacillus subtilis*, to promote heme production (Kwon et al. 2003). The result showed the greatly positive correlation between 5-ALA and heme production. Also, down-regulation of *hem*B and *hem*H genes decreased the metabolic flux from 5-ALA to heme. Moreover, the regulatory of *hem*A and *hem*L, *hem*B, *hem*D, *hem*F, *hem*G, and *hem*H genes on heme synthesis pathway was investigated (Zhang et al. 2015). The regulation of transcription level of genes was also explored by quantitative real-time PCR, in which feedback inhibition of *hem*B by the intermediate PPIX was verified, suggesting that regulation of heme biosynthesis pathway genes is complicated. Zhao et al. confirmed that the C5 pathway obtained superior performance over C4 pathway for heme production, and further adjusted the metabolic flux involved in heme biosynthesis (Zhao et al. 2018).

The strategies for 5-ALA production in *E. coli* via C5 pathway in the past decade are organized in Table 3. A small mRNA, *ryh*B, was applied in *E. coli* to regulate the heme biosynthesis pathway and 5-ALA production (Li et al. 2014). The RyhB is able to reduce iron-binding proteins expression and has been proved to down-regulate transcription level of *hem*B and *hem*H; thus, the 5-ALA accumulation was enhanced to 116%. The regulatory of heme synthesis pathway was explored which relied on the transcriptional regulation of each gene in the pathway (Kang et al. 2011a, b). Kang et al. firstly over-expressed *hem*A from *S. arizona*, *hem*L, and threonine/homoserine exporter *rht*A from *E. coli*, reaching 4.13 g/L of 5-ALA from glucose without any other co-substrate or inhibitor. At first, the up-regulation of *hem*D and *hem*F increased 5-ALA accumulation. Next, the over-expression of *hem*B, *hem*G, or *hem*H caused reduction on most of genes in the pathway, while over-expression of *hem*D resulted in significant increase of ALA. After applying modular optimization for expression cassette, the highest 5-ALA was achieved at 3.25 g/L. The iron was also found to be indispensable for cell growth and heme biosynthesis (Zhang et al. 2016). With the optimized iron concentration and co-expression of *hem*A, *hem*L, *hem*F, and *hem*D, the 5-ALA production achieved 4.05 g/L in batch fermentation. Su et al. employed CRISPRi system to fine-tune *hem*B on heme synthesis pathway (Su et al. 2019). The 6 CRISPRi targeting various regions of *hem*B were selected for down-regulation of the gene by 15, 19, 33, 36, 71, and 80% respectively. The optimized targeting site enhanced fourfold of 5-ALA compared to the original strain, thus providing a new insight into fine-tuning the heme biosynthesis pathway for 5-ALA accumulation.

**Table 3:** Production of 5-ALA via C5 pathway in *E. coli*

Year	Strain	Strategy	ALA titer (g/L)	ALA productivity (g/L/h)	Ref
2011	DH5a	Expressing *hem*A from *S. Arizona* and identify exporter RhtA for ALA	4.13	0.086	Kang et al. (2011a, b)
2014	DH5a	Using small RNA *ryh*B to enhance ALA production	1.5	0.047	Li et al. (2014)
2015	BL21(DE3)	Identify the relation of ALA production and genes on heme synthesis pathway, combinatorial over-express *hem*A, hemL, hemD, and hemF with different copy-number plasmids	3.25	0.108	Zhang et al. (2015)
2015	BL21(DE3)	Co-overexpression of the heme synthesis pathway genes hemA, hemL, hemF, and hemD with the addition of 7.5 mg/L iron	4.05	0.127	Zhang et al. (2016)
2017	W (ATCC9637)	Applying synthetic and strong promoter for hemA from *S. typhimurium* and hemL, deletion of sucA and fine-tune gltA and aceA level	3.4	0.189	Noh et al. (2017)
2019	BL21(DE3)	Optimize hemA and hemL level with RBS engineering, weaken hemB, and strengthen pdxH for PLP synthesis. Finally improve the production in 3-L fermenter	5.25	0.146	Zhang et al. (2019)
2019	DH5a	Fine-tuning hemB by CRISPRi system at various targeting sites and using fed-batch fermentation	1.99	0.046	Su et al. (2019)
2019	MG1655	Integrating multiple copies of hemA-hemL by CIChE in DrecA strain	4.55	0.063	Cui et al. (2019)
2019	Transetta(DE3)	Expressing hemA1 (GTR) and pgr7 (GBP) genes from Arabidopsis thaliana	7.64	0.159	Aiguo and Meizhi (2019)
2020	DH5α	A heme-responsive regulatory system containing a heme biosensor HrtR and CRISPRi was designed to regulate chemicals production while maintaining the intracellular heme homeostasis	5.35	0.111	Zhang et al. (2020a)

The above references represented the great relationship between 5-ALA in C5 pathway and heme synthesis, and the regulation of transcriptional level on the pathway affected both 5-ALA and heme accumulation. Therefore, understanding the overall regulatory mechanism of heme biosynthesis pathway genes is vital for 5-ALA production.

### Other regulations in TCA cycle

The metabolic engineering has been greatly benefited from the synthetic and system biology owing to the abundant advancement as it collaborated with metabolic regulation and has applied to optimize natural chemical production (Jones et al. 2015). Producing maximum chemical is the principal objective for balancing metabolic pathway, while the imbalance occurred in the host causing death to the cell (Yang et al. 2020). Since 5-ALA can be biologically produced via metabolic route in which the TCA cycle and heme synthesis pathway are involved, the regulation and redistribution of metabolic flux are crucial for its production in microorganisms.

Not only the heme synthesis pathway is crucial to 5-ALA accumulation but engineering metabolic flux from the upstream and TCA cycle is also important to its biosynthesis. The carbon flux from glucose was optimized by Noh et al. by over-expressing *glt*A and *ace*A with the deletion of *suc*A (Noh et al. 2017). Moreover, *hem*A from *S. typhimurium* with addition of two lysine codons was employed for its higher activity and stability. However, the deletion of *suc*A resulted in lower biomass and 5-ALA production, which came from the impaired TCA cycle of insufficient energy production. The over-expression of *glt*A was reported to show the negative effect on 5-ALA accumulation but a slight improvement in cell biomass and glucose consumption. Finally, the carbon flux on glyoxylate cycle was optimized by fine-tuning the transcription level of *ace*A, and 3.4 g/L of 5-ALA was obtained after the optimization of culture condition. Moreover, the tuning relationship between heme synthesis genes and 5-ALA production was also provided. Zhang et al. engineered multiple ribosome binding site (RBS) for *hem*A and *hem*L over-expression and replaced the promoter of *hem*B to weaken 5-ALA catabolism (Zhang et al. 2019). Besides, the co-factor PLP for ALAS was synthesized by over-expressing *pdx*H and the 5-ALA production was finally increased to 5.25 g/L with a two-stage fermentation. This is the first report implementing *pdx* gene for PLP enhancement to improve 5-ALA accumulation in *E. coli*. Xue et al. also fine-tuned the supplementation of PLP by integrating *pdx*Y gene and has augmented the 5-ALA synthesis via C4 pathway (Xue et al. 2021). Aiguo and Meizhi introduced *hem*A1 (encoding glutamyl-tRNAGlu reductase) and its stimulator protein encoded by *pgr*7 genes from higher plant *Arabidopsis thaliana* into *E. coli* for overproduction of 5-ALA via C5 pathway (Aiguo and Meizhi 2019). By over-expressing *hem*A1 and *pgr*7 and up-regulating *zwf*, *gnd*, *pgl,* and *rht*A, 7.64 g/L 5-ALA was produced from 10 g/L glutamate and 15 g/L glucose at 48 h. In order to obtain a stable and efficient strain for 5-ALA, chemically inducible chromosomal evolution (CIChE) strategy was carried out by Cui et al. for high gene copy expression in *E. coli* recently (Cui et al. 2019). The expression cassette containing *hem*A from *S. arizona* and *hem*L from *E. coli* was integrated onto MG1655 and successfully reached 98 copies with 2.72 g/L of 5-ALA. After a long-term adaptive evolution and the deletion of *rec*A, the 5-ALA accumulation was improved and achieved 4.55 g/L at 72 h. The glutamyl-tRNA reductase encoded by *hem*A was also reported that its N-terminus played a critical role on its stability control, which has been improved by inserting lysine or arginine residues behind Thr2 (Zhang et al. 2017). A heme-responsive regulatory system was designed to containing a heme biosensor HrtR and CRISPRi for regulation of 5-ALA production as well as maintaining the intracellular heme homeostasis for cell growth (Zhang et al. 2020a). The metabolite-binding affinity of HrtR was modified by heme titration and dynamic simulation. They finally improved the 5-ALA production to 5.35 g/L by down-regulating the PBG formation with the regulatory.

**Fig. 2:**
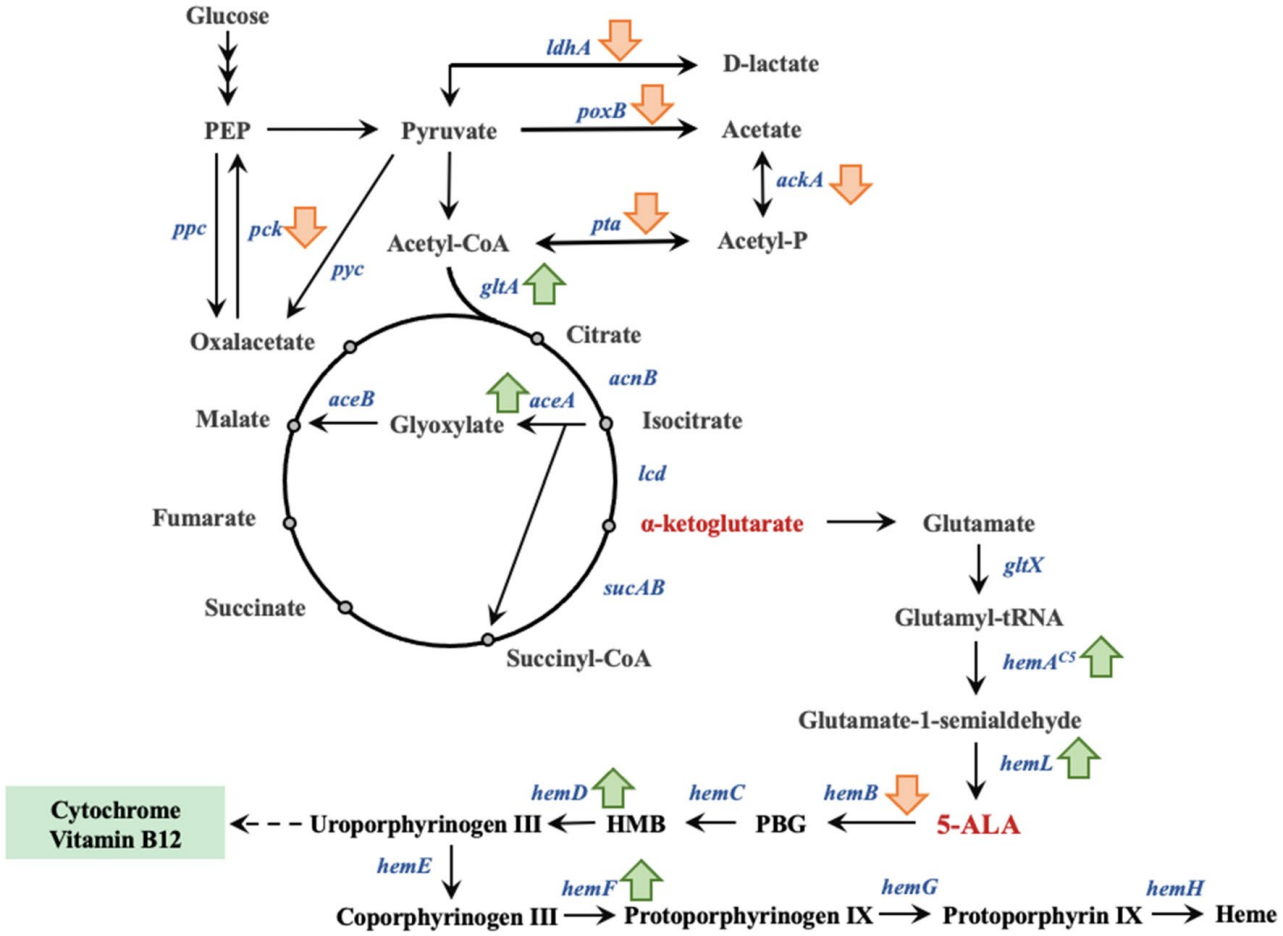
The C5 pathway for 5-ALA and heme synthesis. The up and down arrows beside the gene indicate the up- or down-regulation of the gene which can increase 5-ALA accumulation

The total concept of 5-ALA production from C5 pathway is shown in Fig. 2. Although the production of 5-ALA via C5 pathway is more difficult than via C4 pathway, it is still important to explore the mechanism and regulation on heme synthesis pathway. Without the demand of substrates (i.e., glycine and succinate), producing 5-ALA via C5 pathway may be more economical and ecofriendly.

## Biosynthesis of 5-ALA in different microorganisms

Besides *E. coli*, other organisms have also been studied for more efficient 5-ALA production to achieve different benefits. By applying suitable strains, studies have reported to reach higher 5-ALA accumulation or deal with other issues at the same time. The reports provided the ideas and opportunities for 5-ALA in various organisms and application (Table 4).

**Table 4:** Biosynthesis of 5-ALA in micro-organisms other from *E. coli*

Year	Pathway	Strategy	ALA titer (g/L)	ALA productivity (g/L/h)	Capacity (L)	Ref
*Corynebacterium glutamicum*						
2015	C5	Expressing *hem*A from *S. typhimurium* with codon optimization of *hem*L and increase cell membrane permeability with penicillin G	2.2	0.046	2.0	Ramzi et al. (2015)
2015	C5	Expressing *hem*A from *S. arizona* with mutation and *hem*L from *E. coli*. Added 0.01 mg/L ferrous ion in shaking flask	1.79	0.012	0.05	Yu et al. (2015)
2016	C4	Expressing hemA from *R. sphaeroides* and *ppc* with deletion of penicillin-binding genes	7.53	0.209	5.0	Feng et al. (2016)
2016	C4	Expressing ALAS from *R. capsulatus* and RhtA from *E. coli* in fed-batch fermentation	14.7	0.92	0.05	Yang et al. (2016)
2017	C4	Expressing codon-optimized hemA from *R. sphaeroides* and *gly*A in shake flask with deregulation of L-serine operon	3.4	0.27	0.05	Zou et al. (2017)
2018	C5	Expressing hemA from *S. arizona* with mutation, *hem*L from *E. coli,* and deleting glutamate transporter and using weaker RBS for *hem*B	0.895	0.012	0.025	Zhang and Ye (2018)
2019	C5	Inhibit oxoglutarate dehydrogenase with mutation of OdhI, expressing hemA from *S. arizona* with mutation and RhtA from *E. coli*	2.9	0.06	0.02	Ko et al. (2019)
2020	C5	Modulating the genes involved in 5 co-factor regeneration and control *odh*A expression using auto-inducible. Regulate the RhtA by the two-component system HrrSA in response to heme	3.16	0.049	0.045	Zhang et al. (2020b)
2020	C4	Expressing ALAS from *R. palustris*, using cassava bagasse hydrolysate as carbon source and optimization of *ppc* expression	18.5	0.47	2.0	Chen et al. (2020)
*Rhodobacter sphaeroides*						
2015	C4	Optimizing metal ion to produce biomass and 5-ALA from *R*. *sphaeroides* in wastewater	1.15	0.032	0.5	Liu et al. (2015)
2018	C4	Fe^2+^ effectively enhanced the biomass production and 5-ALA yield of *R. sphaeroides* Fe^2+^ improved ATP production by up-regulating the *nif* gene which enhanced the biomass and ALA	4.02	0.112	0.5	Liu et al. (2018)
*Streptomyces coelicolor*						
2019	C5	Integrating hemA from *R. sphaeroides* into *S. coelicolor* and optimized in flask cultivation	0.137	0.005	0.05	Tran et al. (2019)
*Bacillus subtilis*						
2020	C5	Over-expression hemA and hemL from *B. subtilis*	0.0685	0.0011	N.D	Liu et al. (2020)
*Shewanella oneidensis*						
2020	C5	Showing that higher 5-ALA could be obtained from wild-type *S. oneidensis* than from *E. coli*. Over-expressing *hem*D and *hem*F under T7 promoter	N.D	N.D	0.05	Yi ang Ng (2020)
2021	C4, C5	Applying CRISPRi system to concentrate carbon flux and over-expressing ALAS under T7 promoter	0.207	N.D	0.05	Yi ang Ng (2021)
*Saccharomyces cerevisiae*						
2018	C4, C5	Over-expressing *hemL* from *S. cerevisiae, hemA* from *R. spheroids*, *hemA* and *hemL* from *E. coli* with addition of glycine and succinate	0.526	N.D	N.D	Zhang et al. (2018a)
2019	C5	Over-expressing *HEM1, ACO1,* and *ACO2* from *S. cerevisiae*. Optimization of medium, precursor glycine, and inhibitor levulinic acid	0.004	8.3*10^–5^	0.05	Hara et al. (2019)
2020	C5	Over-expressing *HEM1* to produce 5-ALA by solid-state fermentation process. Reached 225.63 mg/kg 5-ALA dry materials after process optimization	0.063	0.0013	11	Mao et al. (2020)

N.D. means not determined

### Corynebacterium glutamicum

*Corynebacterium glutamicum* is one of the most important microorganisms for amino acid production, which utilizes C5 pathway for 5-ALA biosynthesis. The recent results of 5-ALA production using *C. glutamicum* are summarized in Table 4. Ramzi et al. firstly exploited *C. glutamicum* for 5-ALA production by over-expressing *hem*A from *S. typhimurium* and *hem*L from *E. coli* (Ramzi et al. 2015). Finally, the highest production was achieved at 2.2 g/L under penicillin induced in the fermentation. The 5-ALA synthesis and heme pathway in *C. glutamicum* were discussed by Yu et al., showing the similarity of regulation between *C. glutamicum* and *E. coli* (Yu et al. 2015). *C. glutamicum* was able to produce 1.79 g/L of 5-ALA with co-expression of *hem*A from *S. arizona* and *E. coli hem*L in cooperation with optimal iron concentration. To efficiently convert L-glutamate to 5-ALA via C5 pathway, *hem*A and *hem*L from *S. typhimurium* and *E. coli* were tandemly over-expressed in which glutamate transporter encoded by *ncgl1221* was knocked out to prevent glutamate secretion and weaken *hem*B by replacing its RBS to improve 5-ALA production (Zhang and Ye 2018). Another observation was that the 5-ALA synthesis was improved by inactivating *lys*E and *put*P, which reduced the conversion of glutamate to arginine and proline. Thus, to block the secretion of these amino acids has ensured that glutamate is sufficient for conversion into glutamate and further toward 5-ALA. By the three single deletion strains, it could achieve higher 5-ALA titer compared to strain using original *C. glutamicum* S9114. Ko et al. devoted on the flux redistribution of the TCA cycle toward L-glutamate in *C. glutamicum* (Ko et al. 2019). As a result, the site-directed mutagenesis was applied in oxoglutarate dehydrogenase inhibitor (OdhI) to allow more glutamate accumulation by inhibiting 2-oxoglutarate dehydrogenase complex. The exporter *rht*A from *E. coli* and the induction of trigger for glutamate from ethambutol enabled 2.9 g/L of 5-ALA production finally. Zhang et al. designed HrrSA regulatory system to fine-tune the RhtA expression for 5-ALA export in response to extracellular heme (Zhang et al. 2020b). The *odh*A encoding α-KG dehydrogenase was also modulated by temperature-induced promoter to optimize the expression for higher 5-ALA accumulation. Under precise control of dynamic metabolic engineering, 3.16 g/L 5-ALA was achieved through C5 pathway in this study.

The first study applied C4 pathway in *C. glutamicum* was reported by Feng et al. (2016). The *hem*A from *R. sphaeroides* was codon optimized and expressed in *C. glutamicum* to enhance 5-ALA accumulation. Several genes responsible for acetate and lactate formation were knocked out to centralize the carbon flux (i.e., *ldhA*, *pqo*, *pta*, *ackA,* and *cat*). Moreover, four genes encoding high-molecular-weight penicillin-binding proteins (HMW-PBPs, encoded by *pbp1a*, *pbp1b*, *pbp2a,* and *pbp2b*, respectively) were deleted to increase the permeability of cell wall, and further enhanced extracellular 5-ALA accumulation to 7.53 g/L. Since glycine is an important precursor for 5-ALA synthesis via C4 pathway, the biosynthesis pathway of serine and glycine was remodeled by Zou et al. to improve 5-ALA accumulation based on the multi-gene engineered *C. glutamicum* strain as aforementioned (Zou et al. 2017). The final 5-ALA concentration was 3.4 g/L by over-expressing *ser*B, *ser*C, mutant *ser*A, and codon-optimized *hem*A from *R. sphaeroides*. Out of expectation, deletion of *sdaA* (encoding L-serine dehydratase, which converts L-serine into pyruvate) decreased biomass and 5-ALA titer due to decline the amount of pyruvate. Yang et al. evaluated several ALAS from different sources and observed a codon-optimized ALAS from *R. capsulatus* displaying the best potential (Yang et al. 2016). Although the TCA derivative metabolites showed no significant difference in *sucCD* deficient strain, the 5-ALA production indeed increased. With the co-expression of *E. coli rht*A, the titer of 5-ALA was increased to 14.7 g/L through two-stage fermentation from glucose and glycine. However, Chen et al. found out that the *hem*A from *R. palustris* possessed higher activity in *C. glutamicum* than that from *R. sphaeroides* recently (Chen et al. 2020). The ALAS expression was further optimized for enhanced 5-ALA production via RBS engineering and the phosphoenolpyruvate carboxylase (PPC) was over-expressed to improve biosynthesis of oxaloacetate derivatives. Till now, the highest record of 5-ALA production is applying genetic *C. glutamicum* as the best strain, which produced 18.5 g/L after 39 h fed-batch fermentation (Chen et al. 2020).

### Rhodobacter sphaeroides

*Rhodobacter sphaeroides* is also a suitable host for 5-ALA production since it naturally possesses 5-ALA biosynthesis via C4 pathway and has been reported to have strong activity of ALAS. Liu et al. firstly applied *R. sphaeroides* to produce 5-ALA and remove pollutants in wastewater in 2015 (Liu et al. 2015). The light intensity, pH, and concentration of trace elements were optimized to reach higher biomass, 5-ALA production, and chemical oxygen demand (COD) removal. Finally, 31.8 mg/l/h biomass production rate, 1.15 g/L 5-ALA, and 93.3% COD removal were achieved. Since Fe^2+^ dosage was found to be an important factor to 5-ALA production in according to previous report (Liu et al. 2015), the concentration of Fe^2+^ and its effect were discussed in 2018 (Liu et al 2018). The optimized dosage of Fe^2+^ for *R. sphaeroides* was 400 μmol/L and the mechanism revealed that Fe^2+^ vastly improved ATP production by up-regulating the *nif* gene expression. The increasing ATP could enhance the biomass and ALA yield to 4.02 g/L by supplying sufficient energy. In addition, the *nifA* and *nifU* gene expression displayed high consistency of co-transcription at the optimal Fe^2+^ dosage. The 5-ALA production from the wild-type *R. sphaeroides* was still with limitation even most of the studies used the RsALAS (Liu et al. 2015; Shih et al. 2021).

### Saccharomyces cerevisiae

Apart from bacterium, *Saccharomyces cerevisiae*, a well-known model microorganism used for bioproduction of value-added compounds, has also been applied to produce 5-ALA. The C4 and C5 pathways were both enhanced in *S. cerevisiae* for the first time in 2018 (Zhang and Ye 2018b). By over-expressing *hemL* from *S. cerevisiae, hemA* from *R. sphaeroides*, and *hemA* and *hemL* from *E. coli*, the 5-ALA production reached to 525.8 mg/L with addition of glycine and succinate. Hara et al. improved 5-ALA production by over-expressing *S. cerevisiae HEM1*, *ACO1,* and *ACO2* at the first time in 2019 (Hara et al. 2019). The *ACO2* encoding aconitase was elucidated to be the rate-limiting enzyme in 5-ALA biosynthesis since iso-citrate was strictly limited in *S. cerevisiae*. Therefore, increasing gene expression of *HEM1* and *ACO2* effectively enhanced 5-ALA production by adding 40 mM levulinic acid, an inhibitor to 5-ALA dehydratase (ALAD). The solid-state fermentation (SSF) for 5-ALA was developed by Mao et al., reporting that SSF can be used to efficiently enrich feed food with 5-ALA at a low cost (Mao et al 2020). They achieved 63.8 mg/L 5-ALA production in flask cultivation by over-expressing HEM1 from *S. cerevisiae*. Finally, a titer of 225.63 mg/kg dry materials was achieved within 48 h through SSF after process optimization, exceeding the usual effective dosage reported in animal trials. This is the first report on combining the simultaneous saccharification and fermentation (SSF) and microbial 5-ALA production, which broadens the application of 5-ALA for feeds, but it still needs more efforts to improve the productivity.

### Other genus: *Streptomyces, Bacillus, and Shewanella*

*Streptomyces coelicolor*, a gram-positive bacterium well known for the ability to synthesize antibiotics, is considered to be promising hosts for the production of bioactive molecules by expressing foreign genes. Moreover, *S. coelicolor* possesses better glucose utilization than *E. coli* and *C. glutamicum*. The first report to integrate *hemA* from *R. sphaeroides* into *S. coelicolor* genome to produce 5-ALA via C4 pathway was provided (Tran et al. 2019). Their result showed that glucose and yeast extract had strongly positive effect on 5-ALA accumulation in *S. coelicolor*. After optimization of casamino acid, peptone, malt extract, glycine, and succinic acid, they finally produced 137 mg/L 5-ALA in bioreactor culture. Although several studies stated that *S. coelicolor* was a suitable candidate for chemical production due to its low endogenous protease activity and efficient secretion of products as well as the lack of a strong restriction system (Anné et al. 2014), the difficulties on genetic manipulation made it hard to reach comparable yield of product with *E. coli*.

*Bacillus subtilis*, a generally recognized safe cell (GRAS), was also reported as a host for 5-ALA production via C5 pathway by Liu et al. (2020). The endogenous *hemA* and *hemL* were over-expressed effectively under maltose induction and reached 68.45 mg/L 5-ALA after 60-h cultivation. On the other hand, Yi and Ng also put effort in *Shewanella oneidensis* for 5-ALA production, showing the promising ability of *S. oneidensis* to produce 5-ALA after stepwise engineering. The expression level of *hemD* and *hemF* genes was firstly enhanced under T7 promoter in *S. oneidensis*, achieving 4.96-fold improvement on 5-ALA accumulation (Yi ang Ng 2020). The glycolysis pathway was regulated by CRISPRi system to augment the carbon flux into TCA cycle and increase 5-ALA synthesis with assistance of ALAS from *R. capsulatus,* finally reaching 207 mg/L (Yi ang Ng 2021). To sum up, the 5-ALA production requires effective genetic tools and genomic information for DNA manipulation; thus, *E. coli* and *C. glutamicum* are still favorable for 5-ALA biosynthesis.

## Purification of 5-ALA from bioprocess and the applications

**Fig. 3:**
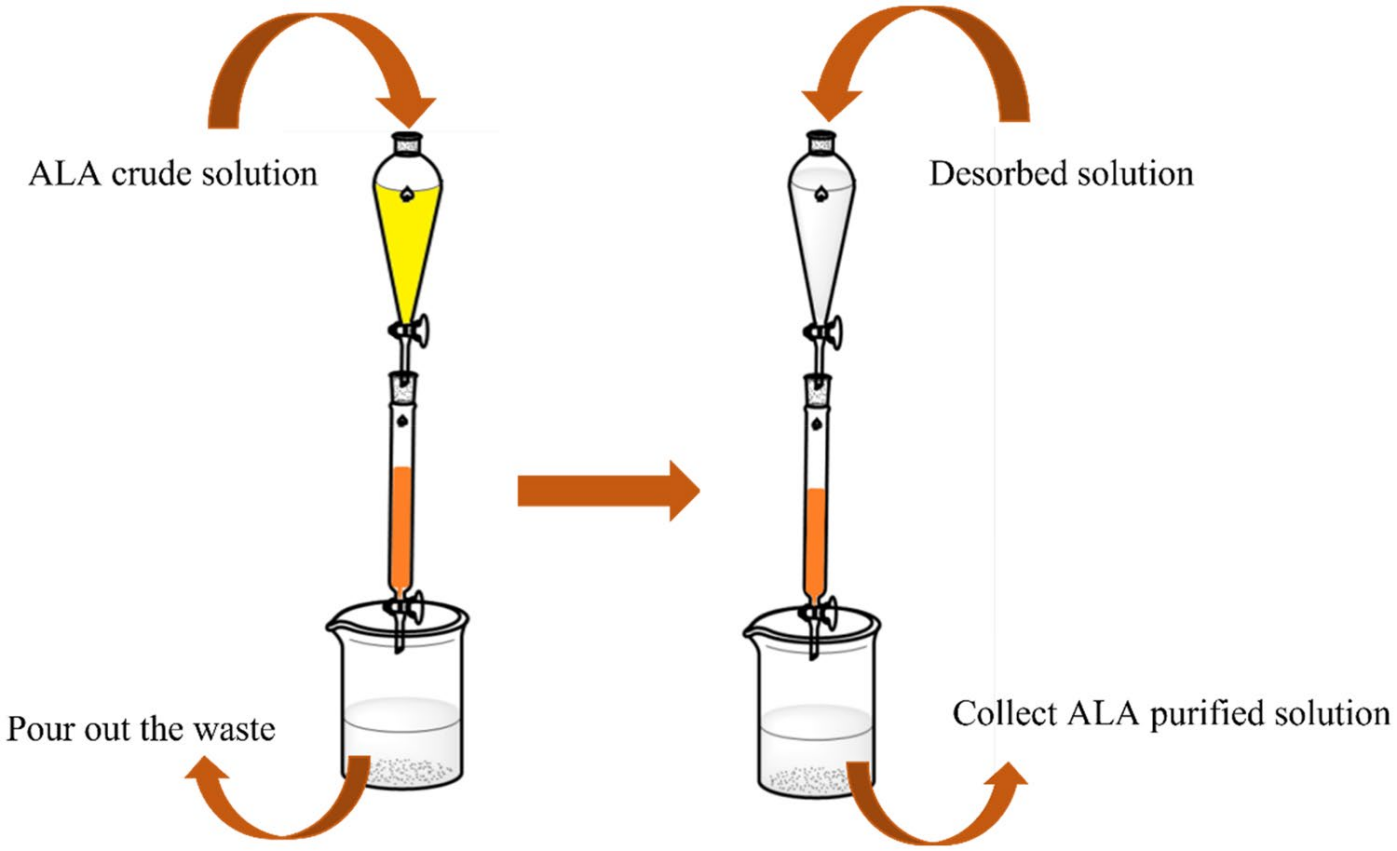
ALA ion exchange process. The resin is filled in the chromatography to adsorb 5-ALA and then washed out by eluent

The biosynthesis of 5-ALA is known as sustainable cost-effective and eco-friendly process; however, the purification is complicated due to the final fermentation broths are containing saccharides, protein, amino acids, organic acids, and metal ions, which are produced during microbial growth. Specifically, glycine may affect the efficiency of crystallization of 5-ALA hydrochloride (5-ALA-HCl) (Okada et al. 2016). Therefore, it is necessary to remove the residual compounds prior to the crystallization by chromatography. Ion-exchange chromatography (IEC) with cation exchange resin (CER) is a common strategy to separate 5-ALA from crude solution (Fig. 3), which is also affected by initial 5-ALA concentration, pH, and eluant (Table 5). Venosa et al. employed a Dowex 50 × 8 (strongly acid hydrogen form) CER to separate 5-ALA from its derivatives with 1 M sodium acetate, reaching 90 ± 4% 5-ALA recovery while only 3 to 9% of the 5-ALA derivatives remained (Venosa et al. 2004). However, suitable condition for separating ALA using IEC for the highest yield is different due to the cultural conditions, such as type of medium and other substances aim at increasing growth. Tripetch et al. optimized the separation method of 5-ALA from *Rhodobacter sphaeroides s*uspension to obtain the highest yield and low cost. First, 2% activated carbon was used in decolorization for 5 to 10 min at pH 5 with 95% efficiency. To purify 5-ALA from the suspension, a strong acid CER Dowex 50WX8 resin was used. The decolorized suspension was washed down through two steps: (1) 1 M sodium acetate (pH4.7); (2) 1 M sodium acetate (pH 3.8). As the result, the recovery was > 70% and the elution time was less than 1000 min (Tripetch et al. 2013). Okada et al. also applied a strongly acid CER Amberlite IR-120B® hydrogen form with 0.3 M ammonia to purify 5-ALA from the culture containing glycine and metabolites, achieving 100% recovery (Okada et al. 2016). Nevertheless, 5-ALA is highly unstable in the alkaline solution after desorption. Hence, 5-ALA solution was directly condensed from 60.3 g/L to 393 g/L by using a vacuum machine, mixed with hydrochloride acid and poor solvent as a precipitation method through dropping the 5-ALA-HCl solution into acetone to obtain crystals of 5-ALA-HCl with 99.5% purity. Lin et al. also condensed 5-ALA solution from 46.8 g/L to 570 g/L with the same approach before crystallization (Lin et al. 2014). The 5-ALA phosphate salt was produced through cooling down at 0 °C with the cooling rate 15 °C/h to form the 5-ALA phosphate crystal, possessing 91.9% recovery and 99.4% purity.

**Table 5:** Purification condition and crystallization for 5-ALA salts

Purification by ion exchange chromatography			
Resin	Eluent (Concentration, pH)	Recovery (%)	Ref
Dowex 50Wx8	Sodium acetate (1 M)	90 ± 4	Venosa et al. (2004)
Amberlite IR-120B	Ammonia (0.3 M)	100	Okada et al. (2016)
Dowex 50Wx8	Sodium acetate (1 M, pH 4.67) followed by sodium acetate (1 M, pH 3.8)	70	Tripetch et al. (2013)

Crystallization				
Salt	Method	Recovery (%)	Purity (%)	Ref
ALA-HCl	Add HCl with 2 times of 5-ALA volume and concentrate the solution to 3 M. Then add aqueous ALA-HCl dropwise to the poor solvents and precipitated crystals of ALA-HCl would be recovered on a filter paper	ND	99.5	Okada et al. (2016)
ALA-Phosphate	Adjust 0.1 M 5-ALA to pH 2.8 ~ 3.2 by adding phosphoric acid. Condense the ALA-phosphate solution to 400–600 g/L before cooling crystallization with 10 ~ 15 °C/h cooling rate and stirring speed 50–200 rpm	91	99	Lin et al. (2014)

Although the microbial production of 5-ALA is abundant, the reports about purification and crystallization of 5-ALA are rare. During the process, the absorption of 5-ALA on resin is efficient, but the low recovery is always caused by long reaction time from desorption and crystallization. Therefore, it is still challenged to purify in acceptable amount of 5-ALA from culture broth.

The 5-ALA with 99% purity is applied in the medical field, while the purity less than 2% is adapted for environmental and agricultural fields (Table 6). The conversion of pure 5-ALA to PPIX in organisms makes it a suitable prodrug for photodynamic therapy (PDT), which is promising treatments to cancer treatment (Nordmann and Michael 2020; Shinoda et al. 2021), dental infections (Amos-Tautua et al. 2019), and condyloma acuminatum (Yang et al. 2020). Moreover, 5-ALA has also applied for the treatment to acne and actinic keratosis, because the generation of porphyrins from 5-ALA boosts the perifollicular inflammatory reaction and activates the expression of keratinocyte-derived IL-8 (Amos-Tautua et al. 2019; Meierhofer et al. 2021).

**Table 6:** Application of ALA in various fields

Field	Application	Ref
Medical	Cancer and tumor localization and treatment	Nordmann and Michael ( 2020), Shinoda et al. (2021)
	Actinic keratosis	Meierhofer et al. (2021)
	Wound infections	Amos-Tautua et al. (2019)
	Periodontal diseases	Amos-Tautua et al. (2019)
	Condylomata acuminata	Yang et al. (2019a, b)
	Acne	Amos-Tautua et al. (2019)
Environment and agriculture	Environmental waters	Amos-Tautua et al. (2019)
	Soil contaminant	Farid et al. (2018)
	Growth regulator for plants	Farid et al. (2018), Wu et al. (2018)
	Biodegradable insecticide	Cheng et al. (2017)
	Yield of crops	Dolmans et al. (2003), Liu et al. (2013)

The study of antibacterial photodynamic therapy (aPDT) used the low concentration of 5-ALA to environmental issue. The accumulated porphyrins from 0.3% of 5-ALA show the different structures with various metal ions and serve as efficient photosensitizer, which destroys bacteria, including sewage bacteriophage, bacterial endospores, and sewage fecal coliforms (Amos-Tautua et al. 2019). 5-ALA is also a biodegradable insecticide as it is a promising nematicide against plant-parasitic nematodes (Cheng et al. 2017). With 1 g/L of 5-ALA (i.e., < 0.1%) and under suitable light exposure, 5-ALA is an effective growth regulator for plants. Wu et al. revealed that 5-ALA remedied the damages of photosynthetic apparatus under salinity by down-regulating the heme content and augmenting the intermediates from the chlorophyll branch (Wu et al. 2018). Based on the advantages of 5-ALA as aforementioned, Farid et al. fed 5-ALA to the sunflowers and increased chlorophyll content and protein expression (Farid et al. 2018).

## Prospective and challenges

As 5-ALA is an important intermediate in the heme synthesis pathway, the accumulation would affect heme biosynthesis which is critical to aerobic metabolism and cell growth. Heme has been widely used in the healthcare and dietary supplement industries as a bioavailable iron-supplying agent (Hoppe et al. 2013). Previous studies have reported the increasing 5-ALA level also showing the positive effect on heme production. The regulation of heme synthesis pathway not only benefits 5-ALA production but also provides prospective strategies for heme accumulation. However, the extraction and purification of 5-ALA and heme from bacterium are difficult. The exporter *rht*A has been widely used for 5-ALA in several studies (Kang et al. 2011a, b; Ko et al. 2019; Yang et al. 2016; Yu et al. 2020; Zhang et al. 2020b), but secretion of heme to extracellular environment was only reported by Zhao et al., using the heme exporter *ccm*ABC (Zhao et al. 2018). Besides heme, the corrin ring of vitamin B12 is also a major tetrapyrrolic product, that is generated from 5-ALA through heme synthesis pathway, which is one of the most fascinating molecules in the medicine (Martens et al. 2002). Interestingly, the glycine riboswitch was used to control 5-ALA production as a regulation between glycine and *hem*B gene to heme (Zhou et al. 2019). Controlling 5-ALA accumulation is a crucial and important direction. Moreover, improving the enzymatic activity via direct evolution with optimal screening platform may accelerate the 5-ALA production in the future (Tan et al. 2020b).

The next generation of green technology is considered in low-carbon footprint and more beneficially to environment. Therefore, the development of molecular biology, genetic, metabolic engineering, or synthetic biology is rapidly extending in bioindustry (Cho et al. 2020). To reach this goal, Tan and Ng have provided a new insight to a high-efficient 5-ALA production with low carbon release (Tan and Ng 2021). In the past ten years, biosynthesis of 5-ALA in microorganisms through metabolic engineering and system biology has drawn an intensive attention due to its high efficiency, sustainability, low cost, and reaching the goal of next-generation biotechnology. Scientists have dedicated to obtaining higher 5-ALA production by a stable strain, which leads to explore various strategies for metabolic regulation and enzymatic engineering. In C4 pathway, in which the 5-ALA production is mainly affected by the expression level and activity of ALAS, the codon sequence and protein structure are critical. Therefore, the optimization of codon and the chaperons play important roles for maximizing the performance of enzyme in *E. coli*. On the other hand, the balancing and regulation of heme synthesis pathway are crucial for 5-ALA accumulation via C5 pathway. Several results showed that there was no significant improvement with the over-expression or down-regulation of genes on heme pathway. Notably, the origins of *hemA* and *hemL* genes used for 5-ALA production were mostly with medium or low copy number.

Exploration of enzyme with higher activity was also feasible for further enhancement of 5-ALA biosynthesis via C5 pathway. In the near future, artificial ALAS with higher activity via C4 pathway will be a promising strategy to accelerate 5-ALA production. In addition, fermentation strategies coupling the feeding of substrates and regulation of metabolic flux could be oriented to more efficient 5-ALA production. In C5 pathway, centralizing carbon flux into heme synthesis pathway is a prospective strategy; however, the metabolic regulation needs to coordinate the 5-ALA and cell growth since the heme synthesis pathway is essential in microorganisms. On the other hand, exploring more different microorganisms for 5-ALA production, especially of lactic acid bacteria which are the major human microbiome, is an alternative approach for direct usage in food (Cho et al. 2020).

5-ALA must be extracted and purified from broth for practical application, especially in medical field; however, the processes are still less described. Meanwhile, instability of 5-ALA during the process is considerable. The operating pH value and temperature may likely result in degradation. 5-ALA belongs to the class of α-amino ketones which easily dimerizes under alkaline conditions (Bunke et al. 2000), and even is unstable in the physiological environment at − 20 °C (Gadmar et al. 2002). Moreover, 5-ALA is a hydrophilic compound and not easily be permeated into cell membrane and periplasm. To solve the above problems, the derivatization of 5-ALA to increasing its lipophilicity, stability in solution, and application field is necessary.

The previous studies of 5-ALA production especially across the *E. coli*, *C. glutamicum,* and some non-typical chassis are summarized in this review. Beyond the fermentation strategies, we highlight the enzyme performance which is critical and must be optimized at the beginning. Chaperone, transporter, and chemical effects according to medium components are also mentioned. Chromosomal engineering is an alternative approach to increase 5-ALA production with higher stability and the regulation of heme biosynthesis pathway by fine-tuning the genes and promoters for 5-ALA biosynthesis are crucial. To broaden the application of 5-ALA, the purification from culture broth is necessary and still requires much improvement. As the 5-ALA and heme are critical for medical applications, more technologies and efforts which including scale-up and product storage will be considered in the future.

## Data Availability

The datasets used and/or analyzed during the current study are available from the corresponding author on reasonable request.

## Abbreviations

5-ALA: 5-Aminolevulinic acid
ALAS: 5-Aminolevulinic acid synthase
AOS: α-Oxoamine synthase
CAI: Codon adaptation index
HMW-PBPs: High-molecular-weight penicillin-binding proteins
PBG: Porphobilinogen
PLP: Pyridoxal phosphate
PPC: Phosphoenolpyruvate carboxylase
PPIX: Protoporphyrin IX
ROS: Reactive oxygen species
PDT: Photodynamic therapy
IEC: Ion-exchange chromatography
